# Study of PVD AlCrN Coating for Reducing Carbide Cutting Tool Deterioration in the Machining of Titanium Alloys

**DOI:** 10.3390/ma6062143

**Published:** 2013-05-24

**Authors:** Natalia L. Cadena, Rodrigo Cue-Sampedro, Héctor R. Siller, Ana M. Arizmendi-Morquecho, Carlos I. Rivera-Solorio, Santiago Di-Nardo

**Affiliations:** 1 Tecnológico de Monterrey, Campus Monterrey, Eugenio Garza Sada 2501, Monterrey 64849, N.L., Mexico; E-Mails: nl.cadena.phd.mty@itesm.mx (N.L.C.); rodrigo.cue.sampedro@itesm.mx (R.C.-S.); rivera.carlos@itesm.mx (C.I.R.-S.); s_dinardo@hotmail.com (S.D.-N.); 2 Centro de Investigación en Materiales Avanzados, S.C. Unidad Monterrey, 66600, Alianza Nte. 202, Apodaca, N.L., Mexico; E-Mail: ana.arizmendi@cimav.edu.mx

**Keywords:** AlCrN films, PVD deposition, cutting tool life, wear resistance, friction coefficient

## Abstract

The manufacture of medical and aerospace components made of titanium alloys and other difficult-to-cut materials requires the parallel development of high performance cutting tools coated with materials capable of enhanced tribological and resistance properties. In this matter, a thin nanocomposite film made out of AlCrN (aluminum–chromium–nitride) was studied in this research, showing experimental work in the deposition process and its characterization. A heat-treated monolayer coating, competitive with other coatings in the machining of titanium alloys, was analyzed. Different analysis and characterizations were performed on the manufactured coating by scanning electron microscopy and energy-dispersive X-ray spectroscopy (SEM-EDXS), and X-ray diffraction (XRD). Furthermore, the mechanical behavior of the coating was evaluated through hardness test and tribology with pin-on-disk to quantify friction coefficient and wear rate. Finally, machinability tests using coated tungsten carbide cutting tools were executed in order to determine its performance through wear resistance, which is a key issue of cutting tools in high-end cutting at elevated temperatures. It was demonstrated that the specimen (with lower friction coefficient than previous research) is more efficient in machinability tests in Ti6Al4V alloys. Furthermore, the heat-treated monolayer coating presented better performance in comparison with a conventional monolayer of AlCrN coating.

## 1. Introduction

Companies and researchers in the field of cutting tools are in the continuous search of coatings capable of improving the behavior of cutting tools for aerospace and biomedical applications (mainly manufactured of carbides) in terms of wear resistance. Depositions of nanoscale and nanostructured multilayered coatings are extensively investigated and developed due to their pronounced strength enhancement, high toughness and excellent wear resistance. In the study of Kao *et al.* [1], TiAlN/CrSiN coatings of multilayered thin films were synthesized and characterized by X-ray diffraction (XRD), scanning electron microscopy (SEM) and transmission electron microscopy (TEM), and the surface roughness was explored by atomic force microscopy (AFM). Also, Vickers and pin-on-disk tests were used to evaluate wear and hardness, respectively. Fox-Rabinovich *et al.* [2] studied mechanical properties of cemented carbide tools coated with TiAlN and AlCrN. Microhardness of the coated tools was measured under different temperatures, ranging from ambient to 500 °C. Also, end milling tests were performed on the coated tools, demonstrating that the life of the TiAlN coating was lower than that of AlCrN, making this composite highly appealing for further research.

Certain changes in deposition, constitution, structure and composition of coating materials make them more suitable for industrial applications. These multilayered coatings are used mainly in cutting tools, implants [3], molds and dies, among other applications. Machining, at considerably high cutting speeds, is known as high-speed machining (HSM) and it is a very accurate technology focused in manufacturing dimensionally precise parts [4]. However, working with HSM implies a great temperature rise in materials which is the major concern in the selection of process parameters. This high cutting temperature generally reduces the tool life and its quality. A wide variety of cutting fluids are used to eliminate the effects of heat and friction, [5] but these may create environmental problems. To avoid pollution and reduce processing cost, new manufacturing dry machining technologies are being performed and, consequently, the physical vapor deposition (PVD) process is used to coat cutting tools [6]. PVD is one of the technologies that improve the tool’s life and productivity. A coated tool can cut faster, reducing the time of production. In the work of Kalss *et al.* [7], the influence of key properties of nitride coatings, in relation to metal cutting, was discussed. Moreover, Wang and Ezugwu [8] investigated the performance of titanium PVD-coated carbide tools, showing improvement under optimum cutting conditions. According to Altuncu and Üstel [9], a proper control of the reactive gas flow rate (nitrogen in this case) as well as the PVD parameters, results in critical growth morphology for a multilayered coating. On the other hand, Hovsepian *et al.* [10] applied PVD technology for coating a TiAl alloy with a CrAlN-coated tool. 

The present research is focused mainly on the development of a new nanostructured coating based on nitride nanocomposite (AlCrN) manufactured by the PVD process, which is a relatively new ternary nitride with high amounts of aluminum with excellent properties at high temperatures, excellent antioxidation characteristics, as well as anti-spalling and debris removal properties for the contact interface [11]. A monolayer heat-treated coating was evaluated and its performance on machinability tests of titanium alloy was compared with the results of an AlCrN-coated tool taken from the literature. 

## 2. Experimental Procedure

A heat-treated monolayer coating of AlCrN (AlCrN-T) was deposited on a tungsten carbide micrograin commercial substrate by physical vapor deposition by cathodic arc (arc-PVD) using a system Bias and Cathodic Arc Evaporation (Oerlikon-Balzers). For the deposition of the coating, a target of Al70Cr30 (at%) alloy was used in a controlled nitrogen atmosphere. The deposition time was adjusted to obtain a layer with a predetermined thickness of 4 μm. The deposition of coating was made under a nitrogen atmosphere to ensure the nitriding of the compound and, next, the sample was subjected to a heat treatment at a temperature of 500 °C during 4 hours under an inert atmosphere. Heat treatment was made with the aim of modifying the coating microstructure, extending the diffusion of nitrogen, and leading to the formation and growth of AlN precipitates. Additionally, heat treatment is very beneficial to eliminate the amorphous phases formed during coating processes and the phases could become more crystalline and also could improve adhesion between coating and substrate. Besides, heat treatment also helps to obtain an improvement of structural integrity and a reduction of stress and fragility in coatings. 

The morphological characterization of the cross-section of the coated and uncoated tool was performed by two scanning electron microscopes: FEI Nova NanoSEM 200 and JEOL JSM-6700F. The elemental chemical analysis was done using an Energy Dispersive X-ray detector (EDXS) with a detection limit of 0.1 wt%. The crystalline structure was characterized by X-ray diffraction (GI-XRD PANalytical X'Pert PRO MRD) with grazing incidence from 20° to 80° and angle of incidence of 0.5°. Hardness tests were conducted by means of a MicroVickers Clemex MMT-X7 indenter equipped with a pyramidal diamond tip Berckovich applying 1 kgf during 10 seconds. 

**Figure 1:**
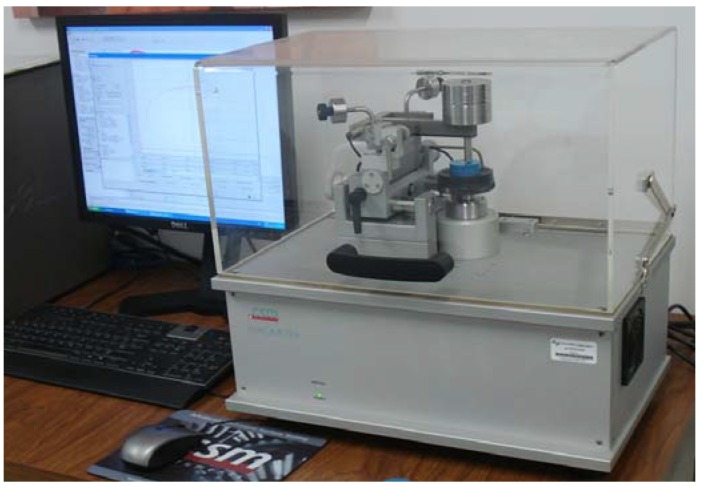
Tribometer using the pin-on-disk technique from CSM Instruments.

**Figure 2:**
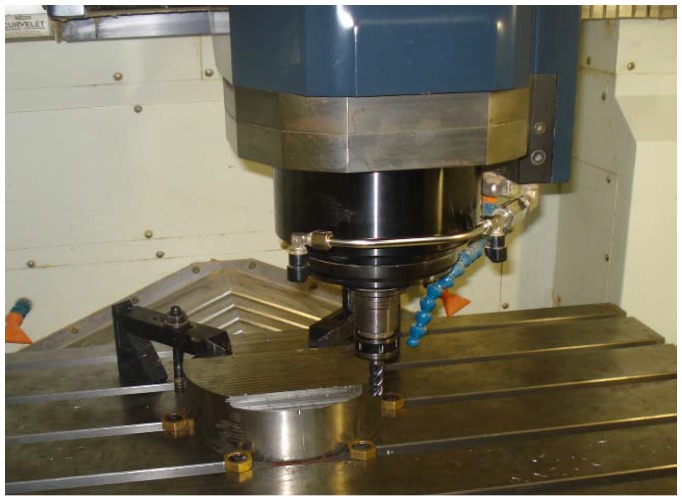
Experimental set up in a three-axis machining center.

Wear tests were carried out on CSM Instruments Tribometer by pin-on-disk test in dry (Figure 1). The values of the coefficient of friction (μ) were obtained directly from the installed Tribox 4.1 software. Sapphire ball with a diameter of 6 mm, roughness R_a_ = 0.02 *μ*m and hardness of 2,300 HV was slid on the WC-Co substrate coated with the AlCrN. Surface roughness measurements were carried out with a Confocal Microscope Carl Zeis Axio CSM-700 on the coating surface; the average value of AlCrN-T sample was R_a_ = 0.86 *μ*m. For the pin on disk test, the sapphire ball was fixed on the load arm and the sample was placed on a rotating disc with a rotating radius of 3 mm. The standard contact loads used were 1, 5 and 10 N. The sliding speed was 0.10 m/s with an acquisition rate of 2.0 Hz and a distance of 1,500 m for the complete test. The temperature during the test was maintained at 26 ± 1 °C with a relative humidity of 30%–40%. Since the wear mass loss values of the samples were inconsistent and the difference between the weight losses was negligible, we rather determined the volume loss values by using a standard test method as indicated in the ASTM G99–05 [12], assuming that there was no significant pin wear using the following equation in accordance with Polok-Rubiniec *et al*. [13]:

(1) $$V=\left(\pi RD^{3}\right)/6r$$

where V is the wear volume (mm^3^); R is the friction radius (mm); D is the wear trace width (mm); and r is the ball radius (mm). Wear rate was calculated directly in the Tribox 4.1 software installed in the tribometer. Machinability tests were performed on titanium alloy (Ti6Al4V) aerospace grade with an end-milling process in a three-axis Vertical Machining Center (Figure 2), with parameters previously determined by screening tests (Table 1) and for accelerated testing purposes. 

**Table 1:** Fixed milling parameters.

Process parameters	Units	Value
Tool fixturing	-	HSK63
Tool diameter, D	mm	12.7
Axial depth of cut, *a_p_*	mm	5
Radial depth of cut, *a_e_*	mm	0.6
Cutting speed, V_c_	m/min	100–150
Feed per tooth, f_z_	mm/tooth	0.04–0.06
Emulsion (refrigerant)	5%	

## 3. Results and Discussion

### 3.1. Substrate Characterization

**Figure 3:**
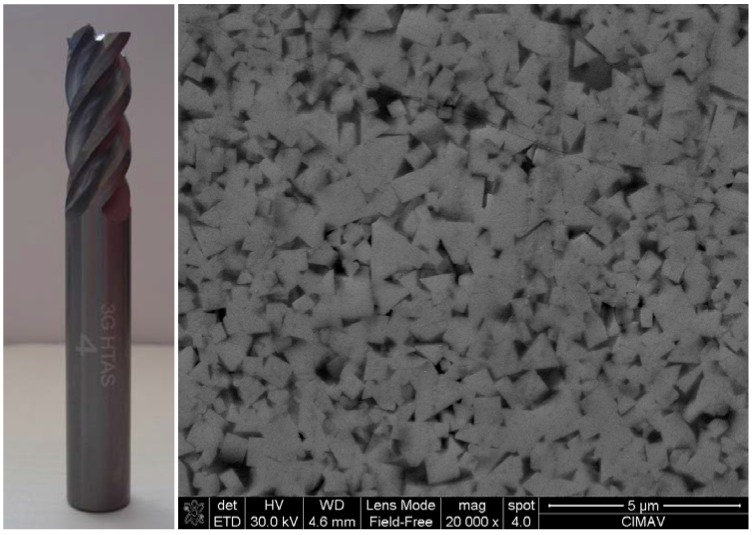
SEM image of the substrate (uncoated) and picture of tool [W: 85.1%at, C: 7.4%at and Co: 7.5%at].

Figure 3 shows a photo of the cemented carbide tool. As it can be observed in SEM photomicrograph, carbide tungsten particles have an irregular and cubic morphology on a scale between 0.2 and 1.4 μm; also a considerable amount of porosity between particles was observed. Tungsten carbide has an average composition in at% of W: 85.1%, C: 7.4% and Co: 7.5%. 

### 3.2. Coating Characterization

**Figure 4:**
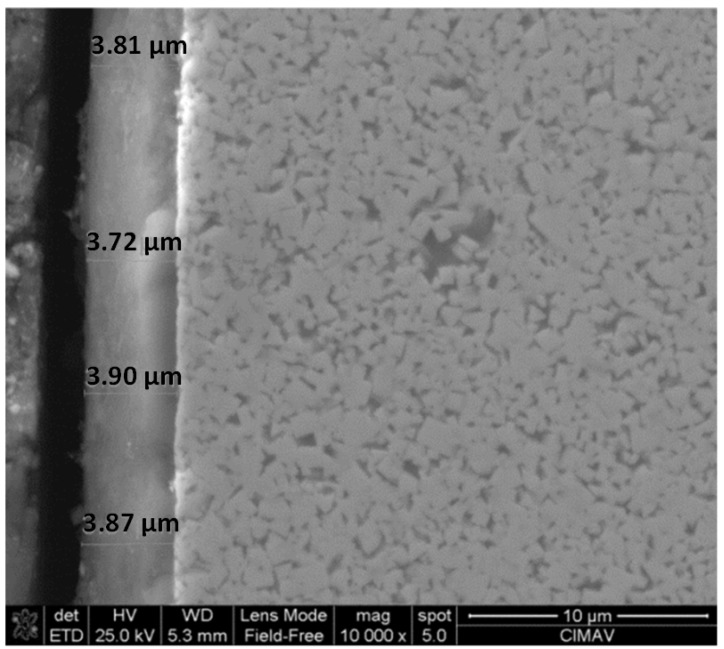
SEM image of AlCrN-T cross section coating [Al 32.31%at, Cr 27.85%at, N 39.84%at].

Figure 4 displays a SEM photomicrograph showing the cross-section of tungsten carbide substrate coated with AlCrN and heat treated. It can be observed that the coating resulted very homogeneously with a thickness around 3.8 μm. The EDXS analysis of the AlCrN-T coating indicates that the chemical composition resulted as follows in at%: Al 32.31, Cr 27.85, N 39.84. 

**Figure 5:**
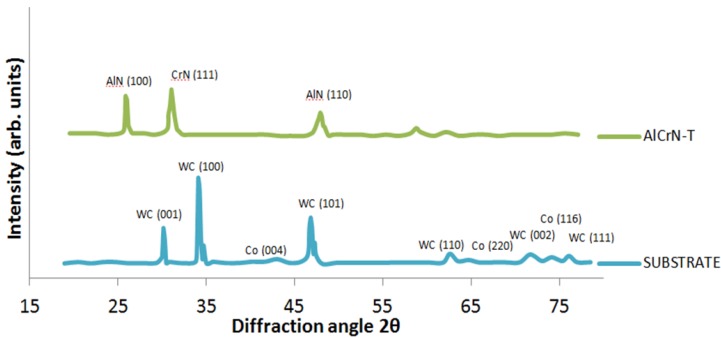
Diffractograms of the AlCrN-T coating and WC-Co substrate.

Figure 5 shows the XRD diffractograms of the coating and substrate. The diffractogram of the tungsten carbide micrograin substrate was joined to the graph to show that the peaks are specifically those of the coating and not a mixture of substrate and film. The AlCrN-T coating shows two structures, CrN cubic corresponding to the Inorganic Crystal Structure Database (ICSD) crystallographic coordinate CrN 01-074-8390, with a preferential peak at the plane (111) and AlN phase with a preferential peak at the plane (100). On the other hand, the heat treatment allowed AlN to recrystallize and CrN to increase its crystal size. Table 2 summarizes the main features of the coated tools found in the last three analyses.

**Table 2:** Main features from XRD characterization of the AlCrN-T coating.

Coating	Main feature	Thickness (μm)	Composition	ICSD	Structure	Planes (hkl)
AlCrN-T	Heat-treated monolayer	4	Al 32.31%at Cr 27.85%at N 39.84%at	CrN 01-074-8390	Cubic	(111)
				AlN 00-025-1495	Cubic	(100) (110)

### 3.3. Wear and Friction Properties

**Figure 6:**
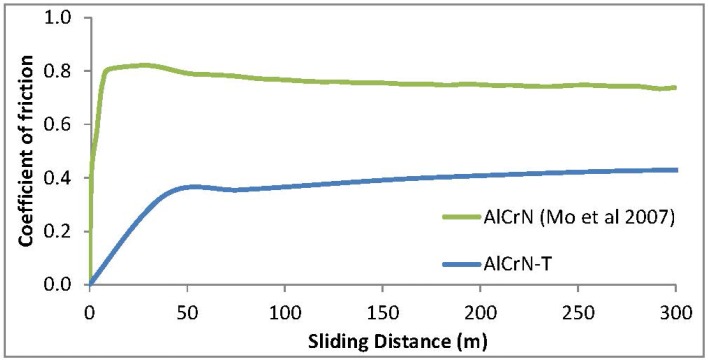
Coefficient of friction of AlCrN coatings using 1 N load.

Figure 6 presents the friction coefficient in function of the distance of the AlCrN-T coating as a result of the pin-on-disk test. Also a coefficient of friction of AlCrN coating from reference [14] is shown for comparative purposes. As it can be seen, the coefficient of friction of the AlCrN-T coating reached the steady stage within 50 m. The average friction coefficient of the heat treated coating was 0.30. It is important to note that coefficient of friction of a monolayer AlCrN coating studied in [14] is higher, showing values of 0.70 in average. 

**Figure 7:**
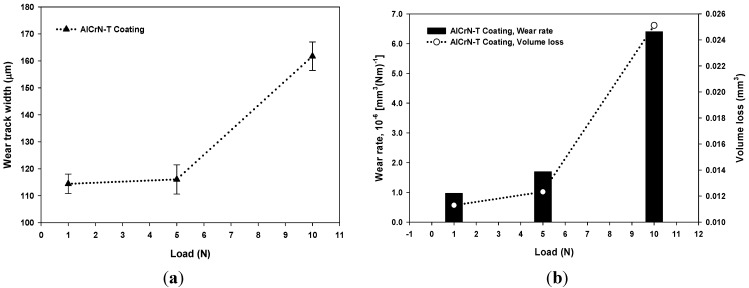

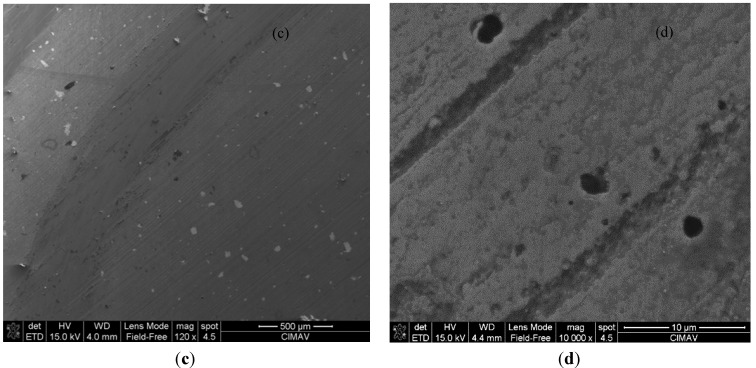
Results of tribological study showing the behavior of AlCrN-T coating: (**a**) wear track width with regard to the applied load; (**b**) Wear rate and volume loss with regard to the applied load; (**c**) and (**d**) Micrographs showing wear track of AlCrN-T coating using 5 N load.

Figure 7 shows the results of the tribological study showing the behavior of AlCrN-T coatings: (a) wear track width regard to the applied load; (b) wear rate and volume loss regard to the applied load; and (c) and (d) wear track of AlCrN-T coating. As it can be seen, the wear track increases with the load. At high applied load, the volume loss and wear rate increase. Using 10 N load, the wear rate is approximately four times compared to low loads; this is because using 10 N load, the pin passes faster beyond the coating approaching the substrate. Using 5 N load, the AlCrN-T coating presents a wear rate of 1.6 × 10^−6^ mm^3^ (Nm)^−1^. It can be seen in Figure 7c,d AlCrN-T presents an adhesive wear and the friction does not detach particles. On the other hand, the surface of coating has a hardness of 1823.3 ± 62 HV.

### 3.4. Machinability Tests

**Figure 8:**
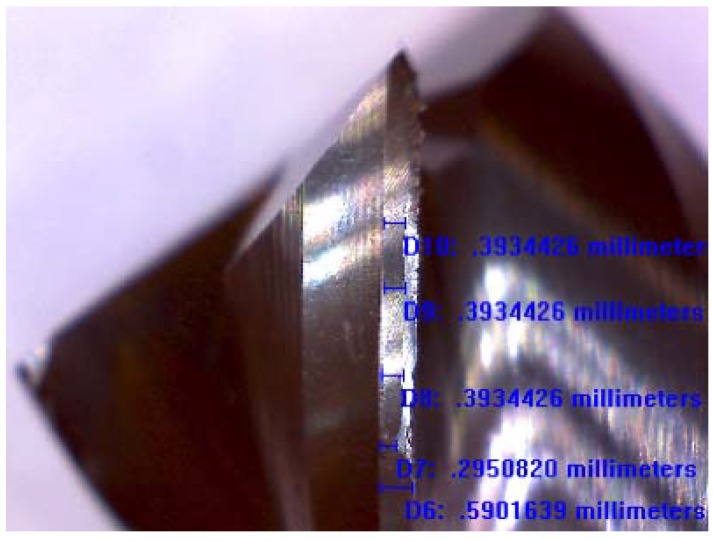
Optical micrograph of flank wear.

Screening machinability tests were carried out over a Ti6Al4V alloy with a control limit of 300 μm of flank wear in the cutting tool, according to the standard ISO 8688-2. Figure 8 shows an example of the measurement of flank wear with an optical microscope and with calibrated scales, considering a previously measured thickness of the flank edge for each tool tested, and a sample machined distance of 5 m. The result of the machinability tests in regards to flank wear of coated tools was 115 μm in average (using a speed Vc of 150 m/min and a feed per tooth of 0.06 mm at a five-meter machined distance). In order to eliminate the influence of the tool run-out in the experimental results, the cutting tools tested had similar run-out values (32–37 μm). 

**Figure 9:**
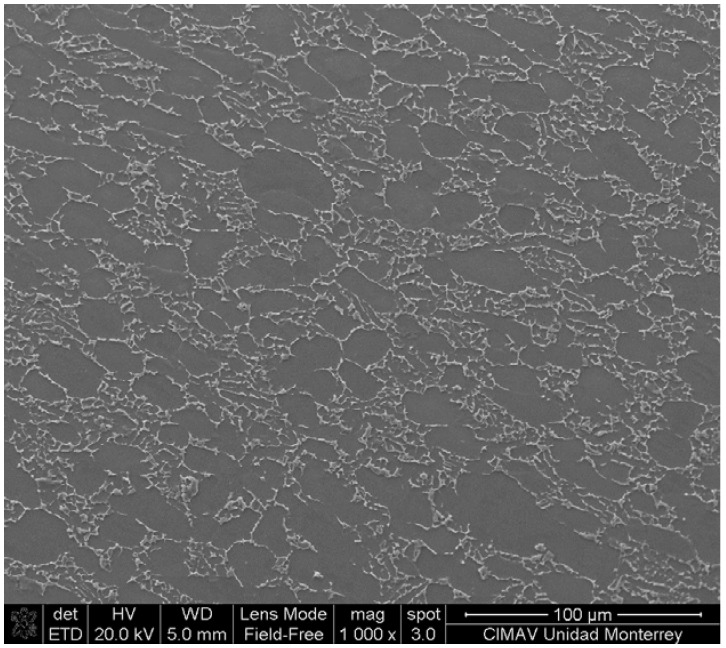
Microstructure of Ti6Al4V substrate that was machined with WC-Co-coated tool (Al 5.64%at, Ti 91.22%at and V 3.14%at).

With the aim of characterizing the capabilities of the deposited AlCrN-T coating in terms of productivity and tool life, further experimentation with Ti6Al4V was performed. This alloy was previously characterized to confirm its chemical composition and hardness. Figure 9 shows a SEM photomicrograph of the titanium alloy and, with EDXS, was confirmed that it corresponds to a Ti6Al4V alloy. The characterization revealed that this material exhibit a bimodal distribution of interconnected equiaxed primary α grains and lamellar (α + β) colonies (transformed β). A careful investigation of the relative distribution of phases indicated an overall primary α content of 60 pct. The average grain size obtained was ~20 μm. The mechanical and thermal properties of this alloy were: hardness 349.4 ± 5.1 HV_500_, tensile strength 950 MPa, yield strength 880 MPa, linear thermal expansion 8.6 µm/m°C, and thermal conductivity 6.7 W/m-K.

**Figure 10:**
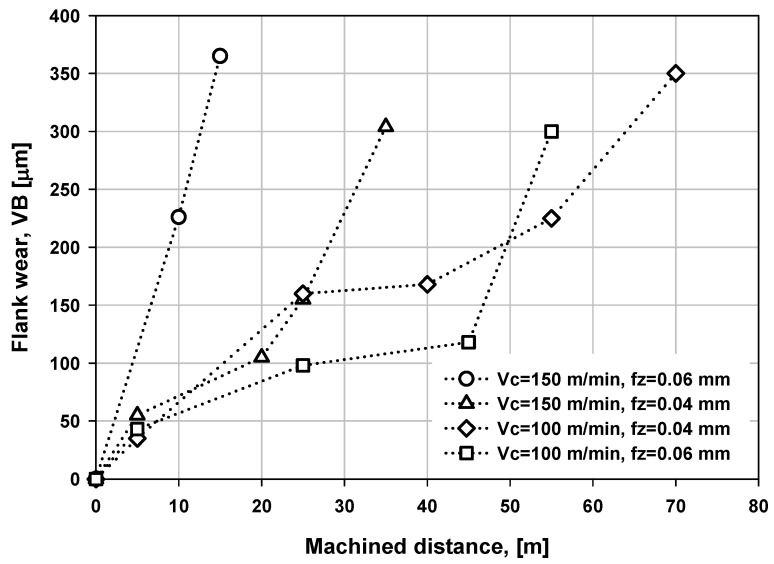
Flank wear of carbide tool coated with AlCrN-T, during machining of Ti6Al4V using different cutting speeds.

In order to evaluate the wear of WC-Co-coated tool machining a titanium alloy, different cutting parameters were used (cutting speed, Vc, from 100 to 150 m/min and feed per tooth, fz, between 0.04 and 0.06 mm). The results of these tests are presented in Figure 10. As it can be seen, with Vc of 150 m/min and fz of 0.06 mm, the flank wear is more pronounced relative to the machined distance. In contrast, the flank wear decreases dramatically when Vc of 100 m/min and fz of 0.04 mm are used. It is also important to note the productivity reached by the operation with the lower levels of cutting speed Vc and feed per tooth fz. This productivity (70 m with a flank wear of 350 μm) is superior than those shown in the literature with the same cutting speed and similar materials (Vc = 100 m/min, end milling of titanium alloys with carbide tool coated), taking as reference the work of Nouari and Ginting [15], in which the amount of material removed was Q = 25210 mm^3^ for 300 μm of flank wear, in comparison with the value reached in this work for the same flank wear (Q = 195,000 mm^3^ or 65 m of machined distance).

## 4. Conclusions 

The AlCrN-T coating deposited by the PVD process showed physical properties with a wide range of applications for manufacture. The structural analysis shows that the heat treatment of AlCrN coating allows recrystallization and crystal growth, enhancing its wear behavior. These characteristics make the coated tools better for cutting applications. 

The AlCrN-T coating presented low friction coefficients and wear rates tested by pin-on-disk, in comparison with previous works [14]. It was revealed that the AlCrN-T coating has a wide potential tribological application under the condition of sliding wear. It took more than 2000 cycles for the AlCrN layer to reach a value of 0.55. 

The machinability study with the coated carbide tool and a workpiece of titanium alloy, presented improved results according to previous research, and it was fully demonstrated that an AlCrN-T coating can be used with acceptable levels of productivity in the machining of aerospace and biomedical components, with adequate process parameters, lubrication and other conditions. Further experimentation should be made in order to demonstrate the viability of other novel coatings (multi-layer) with similar constitutive and heat-treated materials.
